# Are substitution rates and RNA editing correlated?

**DOI:** 10.1186/1471-2148-10-349

**Published:** 2010-11-11

**Authors:** Argelia Cuenca, Gitte Petersen, Ole Seberg, Jerrold I Davis, Dennis W Stevenson

**Affiliations:** 1 The Natural History Museum of Denmark, University of Copenhagen, Sølvgade 83 Opg. S, DK-1307 Copenhagen C, Denmark; 2 L.H. Bailey Hortorium and Department of Plant Biology, Cornell University, Ithaca, NY 14853, USA; 3 New York Botanical Garden, Bronx, New York, NY 10458, USA

## Abstract

**Background:**

RNA editing is a post-transcriptional process that, in seed plants, involves a cytosine to uracil change in messenger RNA, causing the translated protein to differ from that predicted by the DNA sequence. RNA editing occurs extensively in plant mitochondria, but large differences in editing frequencies are found in some groups. The underlying processes responsible for the distribution of edited sites are largely unknown, but gene function, substitution rate, and gene conversion have been proposed to influence editing frequencies.

**Results:**

We studied five mitochondrial genes in the monocot order Alismatales, all showing marked differences in editing frequencies among taxa. A general tendency to lose edited sites was observed in all taxa, but this tendency was particularly strong in two clades, with most of the edited sites lost in parallel in two different areas of the phylogeny. This pattern is observed in at least four of the five genes analyzed. Except in the groups that show an unusually low editing frequency, the rate of C-to-T changes in edited sites was not significantly higher that in non-edited 3^rd ^codon positions. This may indicate that selection is not actively removing edited sites in nine of the 12 families of the core Alismatales. In all genes but *ccm*B, a significant correlation was found between frequency of change in edited sites and synonymous substitution rate. In general, taxa with higher substitution rates tend to have fewer edited sites, as indicated by the phylogenetically independent correlation analyses. The elimination of edited sites in groups that lack or have reduced levels of editing could be a result of gene conversion involving a cDNA copy (retroprocessing). If so, this phenomenon could be relatively common in the Alismatales, and may have affected some groups recurrently. Indirect evidence of retroprocessing without a necessary correlation with substitution rate was found mostly in families Alismataceae and Hydrocharitaceae (e.g., groups that suffered a rapid elimination of all their edited sites, without a change in substitution rate).

**Conclusions:**

The effects of substitution rate, selection, and/or gene conversion on the dynamics of edited sites in plant mitochondria remain poorly understood. Although we found an inverse correlation between substitution rate and editing frequency, this correlation is partially obscured by gene retroprocessing in lineages that have lost most of their edited sites. The presence of processed paralogs in plant mitochondria deserves further study, since most evidence of their occurrence is circumstantial.

## Background

RNA editing is a post-transcriptional process that, in the plant mitochondria, causes a nucleotide change, generally from cytosine to uracil, in mature messenger RNA. It has been estimated that ca. 90% of all encoded genes in the mitochondrial genome of angiosperms are affected by RNA editing [1]. In *Arabidopsis *mitochondria, 441 edited sites are present in coding regions [2], and similar levels of editing have been found in other land plants [3-5]. Editing also occurs but is less frequent in plastids, with only 25 to 30 sites known to be edited in angiosperms [6,7].

Most edited sites are located within protein coding genes, although reduced levels of editing are found in non-coding regions such as introns and tRNAs [[8], and references therein]. It is well established that RNA editing tends to increase conservation of the amino acid composition of homologous proteins across species [9,10]. This is consistent with the fact that editing mostly affects 2^nd ^codon positions, with only a few 3^rd ^codon positions having been observed [2-5].

The mechanisms of RNA editing in plastids and plant mitochondria share a number of characteristics [7]. This, together with the absence of editing in the organelles of green algae, suggests a common acquisition of RNA editing during the origin of land plants [11,12]. However, this conclusion relies on limited data from the green algae.

It has been shown that sequences 20-40 nt upstream and 2-10 nt downstream of an edited cytosine are critical for its recognition as target for editing [[13] and references therein], with a bias towards having a U and an A flanking the edited site at the 5'- and 3'-ends, respectively [2,7]. However, no consensus recognition sequence has been found for all edited sites. Recent studies have proposed that a family of nuclear encoded pentatricopeptide repeat proteins (PPR) are involved in the recognition of sites subject of RNA editing in the organelles [14,15], while it is unclear whether this kind of proteins also carry on the enzymatic reaction. The PPR proteins are encoded by about 450 genes in *Arabidopsis*, suggesting that the entire family diversified and proliferated simultaneously with the establishment of edited sites in land plants [16].

Editing frequency is generally similar among homologous genes across angiosperms [4], and many edited sites are conserved among distantly related groups. However, there is some species-specific diversity in terms of whether a given site either requires editing or the edited version (a T) is encoded in the DNA. Recent observations from a number of plant lineages show surprisingly large differences in editing frequency. For example, there are cases in which all or the majority of the edited sites have been lost in one or more genes [17,18]. It is largely unknown which kinds of processes are responsible for the distribution of edited sites in the mitochondrial genome. In angiosperm mitochondrial genomes, the number of cytosine residues that are edited has decreased over time, being replaced by DNA encoded thymine residues [19]. It has been shown that the C-to-T mutation rate at edited sites is higher than the rate of C-to-T transitions at 3^rd ^codon positions [19,20]. As both substitutions are synonymous, it has been proposed that selection is acting to remove edited sites in plant mitochondria. C-to-T mutations at a site that usually is edited could provide a selective advantage, because this would make editing superfluous and remove any dependency on a number of trans-acting factors and recognition sequences that otherwise need to be conserved [19-21]. This raises the question of how RNA editing evolved in the first place, whether RNA editing could have provided an adaptive advantage during the early stages of land plant evolution [5,8,12] or whether its origin was neutral and driven by genetic drift [22].

Several authors have suggested a possible correlation between substitution rates and editing frequency [16,17,21]. The low substitution rates found in coding areas of plant mitochondria could generate an environment in which it is easier to conserve edited sites by reducing selection pressure. As the substitution rate increases, the mutational load against editing will increase too, as editing relies heavily on conserved recognition sequences [21]. It is noteworthy that lineages with a dramatic increase in substitution rate, such as *Pelargonium *and other Geraniaceae, are associated with a strong reduction in editing [23]. This phenomenon is also observed in species of the genus *Silene *(Caryophyllaceae, [24]). Conversely, higher editing frequencies are found in lineages with a low synonymous substitution rate, such as *Magnolia *[25] and several gymnosperms [17]. Contrary to these examples, there appears to be limited correlation between editing frequency and substitution rates in the Asparagales [18].

No evidence of correlation between substitution rate and editing frequency was found in comparisons of 21 mitochondrial genes in seven land plant taxa [5]. However, this study shows a clear correlation between gene function and editing frequency, hence, a correlation test including all 21 genes simultaneously could confound the effect of gene function and substitution rate over the editing frequency. In order to eliminate the confounding effect of different gene function, gene-specific comparisons need to be done.

Besides C-to-T mutations in the DNA, mitochondrial genes could lose their edited sites by homologous recombination with a cDNA copy [19,26,27]. It has been suggested that in some cases the cDNA copy could be reinserted in a different location within the mitochondrial genome or transferred to the nucleus (creating a processed paralog [26,28]), whereas the original unedited sequence may remain as a functional copy, degenerate into a pseudogene or be completely deleted [26]. However, no evidence of the coexistence of processed paralogs and the original sequence is known in plant mitochondria. The lack of edited sites in *cox*3 and *rps*13 in some lineages of Amaryllidaceae and Iridaceae has been explained by multiple independent incorporation events of processed paralogs into the mitochondrial genome, replacing the original gene [18]. This also applies to the lack of editing in, e.g., *cox*I in *Gingko *and *Larix *[17]. In many of these studies it is impossible to determine whether the C-to-T changes removing the edited sites occurred simultaneously, as expected in the case of gene conversion with a cDNA copy, or whether they have been lost gradually as a consequence of other factors such as changes in mutation rates, increase in selection against edited sites, modifications in the C-to-T fixation rate, or simply random fixation.

To explain why some taxa with a high substitution rate are also lacking edited sites (e.g., *Pelargonium*), an association between retroprocessing and substitution rates has been suggested. It is well known that reverse transcriptase is an error-prone enzyme and consequently a processed paralog may appear to have an increased substitution rate [23]. However, this does not seem sufficient to explain the high substitution rates of some taxa. In addition, it is not evident whether a processed paralog facilitates an increase in substitution rate or if it is the general increase in substitution rate that facilitates the formation of processed paralogs [28].

Recent phylogenetic analyses within the monocots have identified a group within the order Alismatales as one of the lineages with pronounced changes in the editing frequency. In the following discussion we will refer to this group as the "core Alismatales", which includes all families in Alismatales [29] except the Araceae and Tofieldeaceae. Together with a strong reduction in the number of edited sites found in the two mitochondrial genes studied (*cob *and *atp*1, [28]), a number of mitochondrial anomalies have been found in the core Alismatales. This has included a remarkably large number of losses of mitochondrial genes [30], removal of introns in *nad*1 [31], and a likely insertion of processed paralogs [28].

In the present paper we extend the Alismatales data from [28] to include five mitochondrial genes. With these data we aim to 1) explore the correlation between substitution rates and editing frequency, 2) identify possible cases of retroprocessing and explore a possible correlation between this phenomenon and the substitution rates, and 3) test whether the rate of change in edited sites follows the dynamics of C-to-T changes in 3^rd ^codon positions, as expected under neutrality, or if there is any other indication of selection acting to remove edited sites in this group.

We found a significant correlation between synonymous substitution rate and editing frequency for three of the four genes analyzed. Several cases of retroprocessing are hypothesized, in some cases involving the complete gene, which could be indirect evidence of the existence of processed paralogs occurring in the mitochondria. In eight of the twelve families included, edited sites seem to be removed at just a slightly faster rate than the C-to-T changes in 3^rd ^codon positions (difference not significant). This may indicate that in taxa with low substitution rates, edited sites are behaving as effectively neutral, with selection not playing an active roll in purging them from the mitochondrial genome.

## Results

### Phylogenetic analysis

Two most-parsimonious trees (L = 2083, CI = 0.55 excluding autapomorphies, RI = 0.804), were obtained from a parsimony analysis of a combined matrix of five mitochondrial genes (*cob*, *atp*1, exon 2 of *nad*5, *mtt*2, and *ccm*B) and 46 Alismatales taxa (4357 characters of which 647 are parsimony informative). Taxon sampling and GenBank accession numbers are shown in Table S1 in the Additional file 1. The only difference between the trees is in the position of *Stratiotes*, which is placed either as sister to the remaining Hydrocharitaceae or embedded within the family. A maximum likelihood analysis recovered a tree (not shown) identical to one of the parsimony trees, with *Stratiotes *as sister to the remaining Hydrocharitaceae, but also collapsing the clade consisting of Posidoniaceae, Ruppiaceae, and Cymodoceaceae. Because the difference between one of the most parsimonious trees and the likelihood tree has no impact on our analyses, all the subsequent results are based on the former.

**Figure 1:**
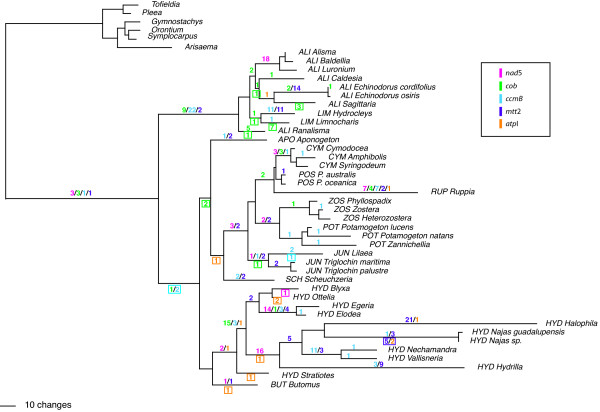
**Mapping of changes in edited sites of five mitochondrial genes**. Mapping of edited sites of five mitochondrial genes (*atp*1, *cob*, *nad*5, *ccm*B, and *mtt*2) on one of the two most parsimonious trees recovered from the combined analysis of all genes (*Acorus *was removed from the figure to save space). Branch lengths reflect the number of changes. For each gene, losses of edited sites are indicated above the branch where the change occurred. Gains of edited sites are indicated in a box below the branch where the change was optimized. Colors represent different genes, according to the box at the right. Family names are indicated next to the taxon name, with the following abbreviations: ALI = Alismataceae, LIM = Limnocharitaceae, APO = Aponogetaceae, CYM = Cymodoceae, POS = Posidoniaceae, RUP = Ruppiaceae, ZOS = Zosteraceae, POT = Potamogetaceae, JUN = Juncaginaceae, SCH = Scheuchzeriaceae, and HYD = Hydrocharitaceae.

All but one of the twelve families included within the core Alismatales are resolved as monophyletic (Figure 1). The exception is Alismataceae, which is resolved as paraphyletic with respect to Limnocharitaceae. In this study we will refer to this clade consisting of Alismataceae and Limnocharitacae simply as Alismataceae *s.l*., unless specified otherwise. In addition, a different editing pattern was consistently found between *Stratiotes *and the rest of the Hydrocharitaceae (see below). Subsequently, we refer to members of this family, excluding *Stratiotes*, as the core Hydrocharitaceae.

### Edited sites and substitution rates

#### Editing in cob

PREP-Mt [10] was used to predict the number of edited sites per taxon and cDNA from a subset of our taxon sampling was used to verify experimentally the predictions from PREP-Mt (Table S2 in Additional file 1). Twenty-five edited sites were found in *cob*, and of those the majority, 16 (64%), are at 1^st ^codon positions (Table 1). Variation in the number of edited sites ranges from 22 in the Araceae to zero in some Hydrocharitaceae (Table S3 in Additional file 1). Only one of the sites found to be edited in RNA sequences was not predicted as edited by PREP-Mt. Neither edited sites at 3^rd ^codon positions nor evidence of incomplete editing were found in *cob*.

**Table 1:** Number of edited sites in 1^st^, 2^nd ^and 3^rd ^codon position and percentage in parentheses

Gene	Edited sites	**1**^**st**^	**2**^**nd**^	**3**^**rd**^
*apt*1	5	2 (40)	3 (60)	0
*ccm*B	44	18 (41)	25 (57)	1 (2)
*cob*	25	16 (64)	9 (36)	0
*mtt*2	30	16 (53)	13 (32.5)	1 (2.5)
*nad*5	25	8 (32)	16 (64)	0

In all genes analyzed, members of Alismataceae *s.l*. and Hydrocharitaceae tend to have fewer edited sites than the remaining members of the order. The dynamic of gain and loss of edited sites was reconstructed in two ways: a) using parsimony to optimize the character change, and b) inferring the edited ancestral codon and predicted amino acid sequence for each node of the tree, and comparing both to observe discrepancies caused by editing. Both approaches indicated that all edited sites present in the most recent common ancestor (MRCA) of the order were lost in the branch leading to Hydrocharitaceae after the divergence of *Stratiotes *(Figure 1). Additionally, nine edited sites were lost in the branch leading to the Alismataceae *s.l*., with subsequent gains and losses arising within this clade. Instances of gain of edited sites were found in *Limnocharis *(7 edited sites gained) and *Sagittaria *(3 edited sites gained).

**Figure 2:**
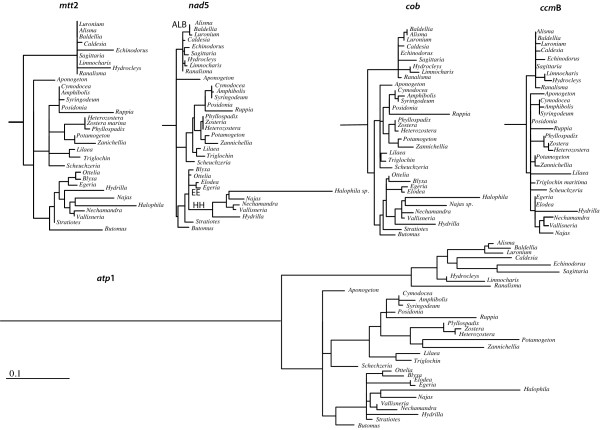
**Synonymous substitution rates (d_S_) for five mitochondrial genes in Alismatales**. Synonymous substitution rates (d_S_) calculated using the program HyPhy ver. 0.9 with the MG94×HKY85_3 × 4 codon model and using one of the two trees obtained by the parsimony analysis for the combined data set. All trees are drawn at the same scale. The outgroup was removed from the figure to save space. Clades marked as HH, EE, and ALB correspond to likely insertions of processed paralogs in *nad*5 (see text).

Synonymous substitution rates (d_S_) were estimated for each branch of the tree. To obtain the absolute synonymous substitution rate (R_S_), the branch length was divided by its time of duration, as obtained by calibrating the tree by penalized likelihood in r8s [32]. However, as single gene d_S _values gave many branches with zero length, we decided to use the distance to the most recent common ancestor of the core Alismatales divided by the age estimated for this node (79.1 Ma) as a taxon-specific estimate of R_S_. A 6-fold difference (P < 0.001) in R_S _was found between the slower (*Triglochin *R_S _= 0.35 × 10^-9^, Figure 2 and Table S4 in Additional file 1) and the faster evolving taxa (*Halophila*, R_S _= 2.13 × 10^-9^). Congruent with this, pairwise relative ratio tests show roughly three groups with significant differences in d_S_: the first is formed by the faster evolving *Halophila, Najas*, and *Ruppia*, the second includes most members of the core Alismatales, and the third includes the slower evolving taxa, such as *Triglochin *and *Stratiotes*.

**Figure 3:**
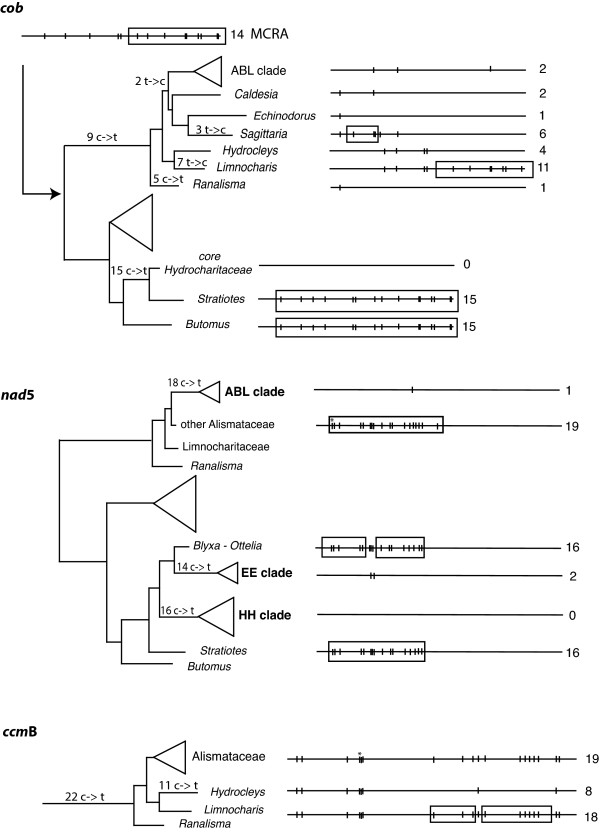
**Changes in the distribution of edited sites of three mitochondrial genes**. Squares represent losses of edited sites from one clade with respect to its sister clade and/or its most recent ancestor.

In order to test whether there is a correlation between substitution rate and the number of changes in edited sites, we performed an analysis of regression through the origin [33,34] and a phylogenetically independent contrast analysis (PIC, [35]). Both methods compare sister clades, which are independent to each other up to the MRCA, and therefore can be included as independent points in a correlation analysis. We found a significant correlation between the d_S _and the number of changes in edited sites in each branch of the tree (r = 0.532, Table 2) when a regression through the origin is carried on. Similarly, the phylogenetically independent contrast analysis shows a significant correlation between the R_S _and the number of changes in edited sites from the MRCA to the tip of the tree (r = 0.477), but not between R_S _and the number of edited sites present in each taxa (r = -0.270, P_(32 df) _= 0.061). These correlations include all species with no edited sites, which may cause an underestimation of the correlation between both variables, because they cannot lose more edited sites, irrespective of their substitution rate (Figure 3). To avoid this, the PIC analysis was repeated removing all the members of the core Hydrocharitaceae where no edited sites were found, leaving only one representative of the non-edited group (either the species with the shortest branch or the one with the longest branch, Table 2). When we do so, all correlations increase considerably, all of them becoming significant.

**Table 2:** Results for the sister group correlations

	d_s _and changes in edited sites	R_s _and changes in edited sites from the MRCA	R_s _and number of edited sites per taxa
*cob*	r = 0.532**	r = 0.477**	r = -0.272
		Long branch	Long branch
		R = 0.735**	R = -0.494**
		Short branch	Short branch
		R = 0.501***	R = -0.180 n.s
*nad*5	r = 0.434*	r = 0.498**	r = -0.489**
		Long branch	Long branch
		R = 0.626***	R = -0.611***
		Short branch	Short branch
		R = 0.572***	R = -0.543***
*ccm*B	r = 0.135 n.s	r = 0.262 n.s	r = -0.187 n.s
*mtt*2	r = 0.603***	r = 0.768***	r = -0.707***

*0.05 > P > 0.01, ** 0.01 > P > 0.001, ***P < 0.001

When the C-to-T substitution rate in 3^rd ^codon positions was compared with the rate of C-to-T change in edited sites, we found that both rates are similar in Aponogetonaceae, Scheuchzeriaceae, Juncaginaceae, Potamogetonaceae, Zosteraceae, Butomaceae, Ruppiaceae, and *Stratiotes *(Table 3). Not surprising, major discrepancies between these rates are found in the Alismataceae *s.l. *and Hydrocharitaceae, where most or all edited sites have been lost.

**Table 3:** Rate of C-to-T change in edited sites (ES) and in 3^rd ^codon positions

	Taxa	*ccm*B		*cob*		*mtt*2		*nad*5	
		ES	**3**^**rd**^	ES	**3**^**rd**^	ES	**3**^**rd**^	ES	**3**^**rd**^
ALI	*Alisma*	**0.52**	**0.025**	**0.79**	**0.037**	0.07	0.063	**0.95**	**0.031**
	*Baldellia*	**0.52**	**0.025**	**0.79**	**0.037**	0.07	0.063	**0.95**	**0.031**
	*Caldesia*	**0.52**	**0.050**	**0.93**	**0.019**	0.07	0.063	0	0.031
	*Echinodorus*	**0.52**	**0.025**	**1.0**	**0.019**	**0.59**	**0.123**	0	0.031
	*Luronium*	**0.52**	**0.025**	**0.79**	**0.037**	0.07	0.063	**0.95**	**0.031**
	*Ranalisma*	**0.52**	**0.025**	**1.0**	**0.037**	0.07	0.063	0	0.031
	*Sagittaria*	**0.52**	**0.025**	**0.86**	**0.037**	0.07	0.063	0	0.031
LIM	*Hydrocleys*	**0.79**	**0.025**	**0.71**	**0.037**	**0.48**	**0.093**	0	0.031
	*Limnocharis*	**0.55**	**0.025**	**0.71**	**0.037**	0.07	0.063	0	0.031
CYM	*Amphibolis*	0.05	0	0.36	0.099	0.07	0.097	0.26	0.062
	*Cymodocea*	0.02	0	0.36	0.079	0.07	0.097	0.26	0.062
	*Syringodeum*	0.02	0	0.36	0.099	0.07	0.097	0.26	0.062
POS	*Posidonia*	0	0	0.14	0.079	0.07	0.097	0.10	0.062
RUP	*Ruppia*	0.16	0.050	0.36	0.206	0.15	0.161	**0.47**	**0.125**
ZOS	*Heterozostera*	0	0.025	0.14	0.140	0.15	0.224	0.17	0.047
	*Phyllospadix*	0	0	0.14	0.120	0.15	0.224	0.17	0.047
	*Zostera*	0.02	0	0.14	0.120	0.15	0.224	0.17	0.047
POT	*Potamogeton*	0.04	0	0	0.099	0.15	0.128	0.17	0.047
	*Zanichellia*	0.04	0.025	0	0.099	0.15	0.159	0.17	0.047
JUN	*Lilaea*	0.07	0	0	0.059	0.15	0.160	0.17	0.047
	*Triglochin*	0.02	0.025	0	0.039	0.22	0.161	0.17	0.047
SCH	*Scheuchzeria*	0.04	0.025	0	0.059	0.07	0.097	0	0
APO	*Aponogeton*	0.02	0.025	0	0.059	0.07	0.096	0	0.017
BUT	*Butomus*	-	-	0	0.059	0.04	0.094	0.05	0.017
HYD	*Blyxa*	-	-	1.0	0.059	0.07	0	0.16	0.017
	*Egeria*	0.15	0	**1.0**	**0.039**	**0.22**	**0**	**0.89**	**0.017**
	*Elodea*	0.17	0	**1.0**	**0.039**	-	-	**0.89**	**0.017**
	*Halophila*	-	-	**1.0**	**0.223**	**0.96**	**0.092**	**1.0**	**0.078**
	*Hydrilla*	0.15	0.025	**1.0**	**0.119**	**0.52**	**0.029**	**1.0**	**0.031**
	*Najas*	0.09	0	**1.0**	**0.139**	**0.29**	**0**	**1.0**	**0.078**
	*Nechamandra*	**0.33**	**0**	**1.0**	**0.119**	**0.30**	**0**	**1.0**	**0.031**
	*Ottelia*	-	-	**1.0**	**0.059**	**0.07**	**0**	**1.0**	**0.016**
	*Stratiotes*	-	-	0	0.079	0.04	0	0.11	0.047
	*Vallisneria*	**0.33**	**0**	**1.0**	**0.119**	**0.30**	**0**	**1.0**	**0.047**

In bold p ≤ 0.001 in the Fisher exact test.

#### Editing in nad5

For this gene, three more edited sites were found in cDNA sequences than were predicted by PREP-Mt. In addition, three sites predicted as edited by PREP-Mt show heterogeneity among taxa in their editing status (e.g., C nucleotides edited in some species but not in others). In particular, three C's edited in *Zannichellia *and *Zostera *show no evidence of being posttranscriptionally modified in *Ruppia *and *Acorus*, and they were excluded from the analysis. This resulted in a total of 25 edited sites in *nad*5, most of them in 2^nd ^codon positions (64%, Table 1). The number of edited sites per species ranged from 22 in Araceae, to zero in members of Hydrocharitaceae (Table S3).

All but one edited site were lost in the *Baldellia - Alisma - Luronium *(ABL) clade, and a reduction in the number of edited sites was also found in *Ruppia *(Ruppiaceae). Instances of ambiguous optimization are found within Hydrocharitaceae, as DELTRAN optimization indicates a parallel loss of 14 edited sites in two branches within the family (Figure 1 and 3). ACCTRAN optimization, on the other hand, mapped these characters as reversals in two branches within the Hydrocharitaceae. This could be a problem of taxon sampling; however, the reconstruction of ancestral codon and amino acid sequences in PAML further supports the parallel loss of those characters, rather than the reversal hypothesis.

An up to 22-fold difference (P < 0.001) in absolute synonymous substitution rate was estimated between *Ranalisma *(R_S _= 0.13 × 10^-9^; Figure 2, and Table S4 in Additional file 1) and *Halophila *(R_S _= 2.88 × 10^-9^). The pairwise relative ratio test shows a significant difference in the substitution pattern of five species of Hydrocharitaceae (*Halophila, Najas, Hydrilla, Nechamandra*, and *Vallisneria*) with respect to the rest of the core Alismatales, which exhibits a homogeneous substitution rate. Weak but significant correlations were found between 1) d_S _and the number of changes in edited sites for each branch (r = 0.434), 2) the R_S _and the number of changes in edited sites from the root to the tip of the tree (r = 0.498) and 3) the R_S _and the number of edited sites per species (r = -0.489). As for *cob*, the correlations increase considerably when members of Hydrocharitaceae with no edited sites are removed from the analysis, and only one representative of the group is left (Table 2).

We found a 3 to 4 times higher rate of C-to-T change in edited sites than in 3^rd ^codon positions (Table 3), except in the two clades where processed paralogs are suspected (e.g., Hydrocharitaceae and the ABL clade, see discussion), or in the remaining Alismataceae, where the rate of change in edited sites is zero.

#### Editing in atp1

For the *atp*1 matrix only five edited sites were predicted by PREP-Mt and corroborated via cDNA sequences. The number of edited sites by taxon varies from zero to three and no edited sites were found at 3^rd ^codon positions. All edited sites in *atp*1 were lost in Alismataceae *s.l*. and in some members of Hydrocharitaceae. *Najas*, *Nechamandra*, *Ottelia*, and *Hydrilla *regained up to three edited sites following complete elimination (Figure 1). *atp*1 showed the highest substitution rate of the genes analyzed in this study (Figure 2). A 4.5-fold rate difference was found between *Aponogeton *(R_S _= 1.30 × 10^-9^) and *Sagittaria *(R_S _= 5.75 × 10^-9^). Given the reduced number of changes of edited sites, no correlation analyses were performed.

#### Edting in ccmB

For some taxa of our data set, no amplification of this region was obtained (Table S1 in Additional file 1). Among-taxon heterogeneity in editing status was found in three positions, and these sites were excluded from the analysis.

*ccm*B shows the largest proportion of edited sites found in this study, ranging from 43 sites in *Aponogeton *to 8 in *Hydrocleys *(Table S3 in Additional file 1). Instances of major losses of edited sites were mapped in the branch leading to Alismataceae *s.l*. (22 sites lost), with a further loss of eleven sites in *Hydrocleys *(Figure 1 and 3). Eleven sites were lost in the *Nechamandra-Vallisneria *clade, and seven in *Ruppia*.

In *ccm*B a 12-fold difference (P = 0.001), in synonymous substitution rate was found between *Posidonia *(R_S _= 0.08 × 10^-9^) and *Hydrilla *(R_S _= 0.99 × 10^-9^). No significant correlation was found either between substitution rate and the number of changes in edited sites in any of the sister group comparisons (Table 2), or between substitution rate and number of edited sites present in each taxa.

#### Editing in mtt2

Thirty edited sites were found in *mtt*2, ranging from 29 in Araceae to one in *Halophila *(Table S3 in Additional file 1). Fifteen of the 30 edited sites found in cDNA sequences of *mtt*2 were either not predicted by PREP-Mt or predicted with a threshold value lower than 0.6. For *mtt*2 the most dramatic losses of edited sites were found in *Halophila*, *Echinodorus*, *Hydrocleys*, and *Hydrilla*, where 21, 14, 11 and nine sites were lost, respectively (Figure 1). Edited sites were regained in *Najas *(five edited sites) only. The relative ratio test for d_S _in *mtt*2 shows an accelerated substitution rate for *Halophila *(R_S _= 2.2 × 10^-9^) with a 3.6-fold difference compare to the slower evolving taxa such as *Stratiotes *(R_S _= 0.6 × 10^-9^, P < 0.001). With the exception of Hydrocharitaceae, *Echinodorus*, and *Hydrocleys*, the rate of C-to-T change in 3^rd ^codon positions is roughly the same as the rate of change in edited sites (Table 3). A significant correlation was found between 1) d_S _and the number of changes in edited sites for each branch of the tree (r = 0.603), 2) R_S _and the number of changes from the MRCA to the tip of the tree (r = 0.768), and 3) the R_S _and the number of edited sites present in each taxa (r = -0.707, Table 2).

## Discussion

### Phylogenetic relationships in the core Alismatales

A robust phylogeny and a dense taxon sampling are fundamental to our understanding of the dynamics of edited sites. However, no consensus exists concerning the phylogenetic relationships among the twelve families that constitute the core Alismatales. The few analyses explicitly addressing this issue are based on a single chloroplast gene, *rbc*L [36,37], or on a combined analysis of *rbc*L and morphological data [38]. Perhaps the most obvious disagreement between our phylogenetic hypothesis and those previously published is in the position of the Alismataceae *s.l*. In our analyses this clade is sister to the remaining core Alismatales, with strong bootstrap support (Additional file 2), whereas the *rbc*L data place it as sister clade to Butomaceae and Hydrocharitaceae [37]. This difference is unfortunate, since the Alismataceae *s.l.*, together with the Hydrocharitaceae, has experienced profound changes in the number of edited sites. However, even the postulated monophyly of Hydrocharitaceae, Butomaceae, and the Alismataceae *s.l*. cannot explain the reduction in number of edited sites as a single event, since most of the changes have taken place only in selected clades within the affected familes. Therefore, regardless of the relationships among these families, we are confident that the dynamics of change in edited sites have been inferred correctly.

The other problematic area of our phylogeny is the placement of *Ranalisma*. Single-gene analyses (not shown) show incongruence in the placement of this genus, and its place in the combined tree is in disagreement with previous studies and with the established classification. When *Ranalisma *is excluded from the analysis, the reversion of seven edited sites of *cob *that were re-gained in *Limnocharis*, are optimized as losses in *Hydrocleys*, which is more in agreement with what we know about the dynamics of editing sites and with the results from other genes.

### Characteristics of edited sites in Alismatales

Although most edited sites are generally present in 2^nd ^codon positions [2-4,8], this is not true for all genes included in this study. In *cob *and *mtt*2, the majority of edited sites are in fact present in 1^st ^codon positions (60 and 53%, respectively). In agreement with other studies [1,5] editing led to an increase in hydrophobicity of the encoded protein. This increase in hydrophobicity is more pronounced in codons with editing in the 2^nd ^position.

The amino acid changes inferred from editing at 2^nd ^codon positions in the core Alismatales correspond to those inferred for *Arabidopsis thaliana, Beta vulgaris*, and *Oryza sativa *[5], with Ser to Phe (34%), Ser to Leu (29%), and Pro to Leu (25%) being the most common. In contrast, replacement of Arg with Trp, by far the most common change in 1^st ^codon position in other taxa [5], accounts for only 14% of changes in the core Alismatales, where the most common changes were from Pro to Ser (30%), His to Tyr (24%), and Arg to Cys (24%). It is not clear whether this difference has any biological significance or if it is a spurious pattern related to the reduced sample size of our analysis as opposed to the broader analysis of Jobson and Qiu [5].

### Dynamics of gain and loss of edited sites

In this study we refer to sites as being edited when DNA-encoded cytosines undergo post-transcriptional modification, resulting in uracil in the mature mRNA. At the DNA level, we consider all changes in which an edited cytosine is replaced by thymine as losses of editing, because this editing is no longer needed to synthesize the correct protein sequence. Another manner of losing an edited site is by a C-to-A or C-to-G change at an edited position, resulting in a non-synonymous change in the encoded protein. We are aware that both scenarios are oversimplifications. Even when the editing mechanism is no longer needed at a particular site (simply because the C subject to editing is not present), this does not necessarily imply that the mechanism itself is lost. Editing relies heavily on interactions between protein factors and recognition sequences upstream and downstream from the edited site. Therefore, it is plausible that, as long as the recognition sequences are conserved, a particular site will not lose the ability to be edited, even though the C has been substituted by another base. This leads us to believe that if a back mutation recovers the original C, this C may possibly still be edited, though this may not always be true. In our analyses ca. 29 T to C reversions were found at editing sites. Although we were unable to confirm their editing status by comparison to cDNA sequences in all cases, we did find heterogeneity between the editing status at some sites. In all cases where heterogeneity among taxa was detected in the editing status, the edited site was removed from the analysis. However, it remains a possibility that we are still overestimating the number of gains of edited sites, because not all reversions were checked against cDNA data to ascertain their editing status.

Consistent with other studies [18,25,28], large differences in the number of edited sites were found among the taxa examined here. Both Alismataceae and Hydrocharitaceae tend to retain fewer edited sites than the remaining members of the Alismatales. Parallel losses were found for both families in at least four of the five genes analyzed, whereas in *atp*1 there are too few instances to draw any conclusion. Reduction in the number of edited sites does not appear to be a process that affects the four genes simultaneously, as each coding region has lost sites in different branches within each family. One of the most problematic branches on which to optimize characters was the branch leading to Alismatales. This ambiguity is attributable to a lack of edited sites in *Acorus *coupled with extensive editing in other members of the outgroup. Further optimization problems were found in *nad*5 within Hydrocharitaceae. A large number of edited sites were lost in this family; however, the precise branch(es) on which a site is lost is difficult to establish. DELTRAN optimization favors parallel loss of edited sites in two branches within the family (Figure 2 and 3), and the same result is obtained by reconstruction of ancestral estates in PAML. ACCTRAN optimization favors a less likely scenario where edited sites are lost at the base of the core Hydrocharitaceae, and secondarily gained in the *Blyxa-Ottelia *clade. Although it has been shown that loss of edited sites occurs more easily than gain [19], we cannot reject the possibility that a reversal may have occurred. A denser taxon sampling within the Hydrocharitaceae may help to unambiguously reconstruct the dynamics of edited sites within the family.

### Changes in edited sites compared with C to T changes in 3^rd ^codon positions

Several authors [19,20] have been suggested that a C at an edited site is not neutral, but has a selective disadvantage with respect to a T in the same position. The presence of edited sites may represent a "burden", since they are dependent on specific recognition sequences and trans-factors that need to be conserved to maintain editing activity. If a mutation re-establishes a genomically encoded T, it may be favored by selection, because dependence on the complex editing machinery is eliminated. These observations are complicated by the observation of reversions or re-acquisitions of edited sites. A strong selective disadvantage would render reversions extremely rare, making it unlikely that up to seven reversions occurred rapidly in some branches of our phylogeny. Although some of the inferred reversals could be an artifact of incomplete taxon sampling or errors in the phylogenetic reconstruction, this is unlikely to be the case for all of them. A large number of mutations restoring an editing site would suggest that a C in a particular position is in some way advantageous.

The idea of selection against editing sites has been supported mainly by a number of analyses showing that the rate of C-to-T substitution at edited sites is significantly higher than the rate of C-to-T change at synonymous positions [19,20]. Edited sites in mitochondrial genes undergo from four to 15 times more C-to-T replacements than C residues at synonymous non-edited 3^rd ^positions [19]. The elevated substitution rate at edited sites is not a consequence of hyper-mutability of DNA motives coincidentally associated with editing. It is, in fact, quite the opposite, since edited sites are usually embedded in regions with low mutation rates [7,20].

Similarly to other studies [19,20], we compare the rate of C-to-T mutations in edited sites with the rate of C-to-T mutations in 3^rd ^codon positions. In most lineages (about 8 of the 12 analyzed families), the rate of C-to-T change in edited sites is not significantly different than the rate of C-to-T change in 3^rd ^codon positions. This may indicate that, in at least a subset of our taxon sampling, edited sites are behaving in a neutral fashion, with no evidence of selection acting to remove them from the genes. It is noteworthy that mutation rates tend to be lower in all of the taxa where the rate of removal of edited sites can be explained exclusively by the synonymous rate of C-to-T mutations. This could further support the hypothesis that at higher substitution rates edited sites become "a burden" to the genome, whereas at low substitution rates this "burden" is so small that edited sites behave as practically neutral. However, we cannot rule out the possibility that this is a spurious pattern caused by the small number of substitutions observed in our dataset.

An additional caveat of comparing the rate of change in edited sites with the rate of change in 3^rd ^codon positions is the assumption that the latter changes are effectively neutral. It is known that edited sites conserve a *cis*-element that is most likely independent of codon location. This could decrease the rate of C-to-T change in 3^rd ^codon positions, since many of those positions may need to be conserved for maintenance of *cis*-elements. This may result in the comparison of a potentially neutral change in the edited site to 3^rd ^positions restricted from C-to-T changes. This bias will be greater in densely edited genes, and it may hide positive selection for C's at certain edited sites. In addition to the recognition of *cis*-elements, differences in codon preference could also be acting to restrict the rate of change in 3^rd ^codon positions in some groups [39].

In contrast to what is found in the other families, the frequency of C-to-T change at edited sites in the core Hydrocharitaceae and some Alismataceae was up to ca. 80 times greater than the frequency of similar changes in 3^rd ^codon positions (Table 3). In fact, in many Hydrocharitaceae all C-to-T substitutions occur exclusively at edited site for four of the five genes analyzed. Two different processes may affect the number of edited sites in these taxa: 1) an accelerated substitution rate and perhaps stronger selection against editing, and 2) retroprocessing, meaning gene conversion with a cDNA intermediary [26-28].

### Likely instances of gene conversion or insertions of processed paralogs

A non-random distribution of editing sites has been shown in a number of genes in the plant mitochondrion [27]. This means that in coding regions groups of edited sites are separated by gaps with limited or no editing. It has also been shown that the distribution of edited sites in some genes corresponds to functional domains of the protein, where selection will favor conservation of editing [1]. Therefore, edited C's outside the structural domains will more easily lose the ability to be edited, as changes in the amino acid composition will have a smaller impact on protein function. However, this hypothesis does not explain the simultaneous loss of edited sites in large regions of the gene. To explain this, several authors have suggested the occurrence of gene conversion with a cDNA copy (e.g., retroprocessing [24]) removing adjacent edited sites and replacing them with the edited sequence [27]. As with many other processes affecting the dynamics of editing, the lack of empirical evidence is manifest. Nothing is known about the sizes of contiguous stretches that retroprocessing could be modifying, or about the recurrence of such events.

Correlations between the number of changes in edited sites and d_S _in *nad*5 highlight three instances where the elimination of edited sites is very high irrespective of the synonymous substitution rate (indicated in Figures. 2 and 3 with the names EE, ABL and HH). We believe that the rapid loss of 14 (EE), 16 (HH), and 18 (ABL) edited sites in these clades could be caused by retroprocessing. One single event of gene conversion could explain the complete elimination of all edited sites in the HH clade and the removal of 17 of the 18 edited sites in the ABL clade (Figure 3). Only in the HH clade is accelerated substitution found. It has been suggested that the insertion of a processed paralog could affect the mutation rate, because the reverse-transcriptase needed to generate the cDNA copies is an error-prone enzyme [23]. However, an insertion of this sort is a single occurrence; it would only have the possibility of introducing a large number of mutations in the apparent ancestor of the group (in which the retroprocessing occurred). Such an occurrence would not explain why all members of the HH clade still show an accelerated substitution rate. It is also unclear how this process could generate an accelerated substitution rate in one clade, but not in others where retro-transcription also may have occurred. The increase in substitution rate in a processed paralog has been associated with its reinsertion into the nucleus [26], but we do not accept this explanation primarily because some edited sites have been regained in the clade (albeit their editing status has not been confirmed by comparison to cDNA copies), and secondarily, because the *nad*5 gene of these taxa still possesses at least one intron. An alternative possibility is the insertion of a cDNA copy (a processed paralog [28]) causing partial gene duplication, and thereby relaxing the selection pressure on one of the copies. However, no evidence of gene duplication or a significant selective shift towards neutrality was found in the group, although more thorough tests need to be performed.

In *cob*, the insertion of a processed paralog is proposed to have occurred in the core Hydrocharitaceae, removing all edited sites in the group [28]. This conclusion is confirmed by our analyses (Figures. 1 and 3). Together with the loss of all edited sites, a single synonymous substitution was found in this branch. In contrast, its sister clade (*Stratiotes*) showed seven synonymous substitutions, but no change in edited sites. A slight acceleration in the substitution rate seems to have occurred in this group, but this occurred after the edited sites were removed, and only in more terminal branches such as *Halophila*. In the Alismataceae clade the situation is less clear. In this particular branch the frequency of C-to-T changes at edited sites over synonymous substitutions is up to 30:1, indicating either very strong selection against edited sites, several events of retroprocessing, or gene conversion with a partially edited cDNA copy [40].

In *ccm*B, 22 losses of edited sites were found in the branch leading to the Alismataceae *s.l*.; however, the losses occurring in this branch seem to be evenly distributed along the gene with the exception of eight edited sites lost in a section of 122 nt, and five lost in a section of 21 nt (Figure 3). Thus, even if the loss of 13 edited sites is explained by two gene conversion events, there are still nine others that cannot be easily explained by this phenomenon. This explanation assumes that retroprocessing affects large stretches in the gene. However, recent evidence suggests that "microconversion" [24] affecting only one or few contiguous sites could be acting to actively eliminate edited sites.

Similarly, the loss of eleven edited sites in *Hydrocleys *could be explained by one gene conversion event that occurred in the last section of 287 nt at the 3' end of *ccm*B.

### Correlation between substitution rate and number of edited sites

For three of the four genes analyzed, we found a correlation between synonymous substitution rates and the number in changes in edited sites per branch in the phylogeny. Except for *ccm*B, this correlation is consistent with the observation that taxa with higher R_S _tend to have suffered major changes in the number of edited sites and have fewer codons that need to be edited (Table 2). As expected, correlations between substitution rates and the number of edited sites increase considerably when cases of suspected insertion of cDNA copies are removed from the calculations, as in members of Hydrocharitaceae in *cob *and *nad*5. An inverse correlation between substitution rates and editing frequency has been suggested by several authors [16,21,23,41], together with the idea that plants displaying an unusual frequency of editing could be those exceptional in having either very high or low rates of synonymous substitution [19]. Our analysis shows that heterogeneity in editing frequency may be found in taxa without exceptional substitution rates. Lynch et al. [21] suggested that the effect of selection against edited sites is only relevant under higher mutation rates, whereas genomes with lower rates would be prone to high frequency of editing, reducing the associated mutation load [16]. Although heterogeneity in substitution rates is found within Alismatales, the differences between fast and slowly evolving taxa are almost negligible when compared with other groups (e.g, Geraniaceae or Caryophyllids). However, we still found that taxa with lower mutation rates tend to have elevated numbers of edited sites. The correlation found between substitution rates and number of edited sites is obscured by other processes such as genetic drift, positive selection, and retroprocessing. In some cases, the presence of taxa that do not conform with this pattern, viz. taxa with reduced editing frequency and low substitution rate, may be explained by gene conversion or insertion of a processed paralog. In other cases, however, retroprocessing is not a useful alternative to explain low substitution rates coupled with low editing frequency, i.e., when the editing sites are lost randomly across the gene, and not in contiguous stretches. The opposite condition is also found, e.g., in taxa with a high substitution rate and high levels of editing (e.g., *ccm*B in *Lilaea*). The strongest correlation between d_S _and editing frequency was found in *mtt*2. Substitution rates in this gene are considerably higher in taxa that have lost most of their editing sites. This makes it difficult to determine whether the lack of most edited sites is a consequence of the high substitution rate (e.g., in *Halophila*) or whether gene conversion has occurred, and if accelerated substitution rate is a consequence of this phenomenon instead.

## Conclusions

In this paper, we have focused on the effect of substitution rates on the rate of change of edited sites. A correlation between the synonymous substitution rate and editing frequency has been found; however, this correlation is weak and is diminished by recurrent gene conversion or insertion of processed paralogs. Additionally, selection does not actively remove edited sites in nine of the 12 families of the core Alismatales. In the remaining three families an up to ca. 80 times increased rate of loss of edited sites with respect to C-to-T changes in non-edited 3^rd ^codon positions was found, indicating either that selection against editing sites is particularly severe in these lineages, or that a different type of process (e.g., retroprocessing) is particularly prone to occur in these taxa. Why retroprocessing has occurred repeatedly in the same groups is something that needs to be explored.

Although the effects of substitution rates, selection and/or gene conversion in shaping the dynamics of editing sites in plant mitochondria is still an open question, it is possible that another type of nuclear mechanism is directly involved in the evolution of edited sites. Recent evidence indicates that a particular class of pentatricopeptide repeat proteins (PPR) is involved in a number of processes within plant mitochondria, and that their proliferation roughly corresponds with the number of edited sites present in different lineages [[42] and references therein]. Hence, an increased understanding of the role of PPR proteins may hold the key to a better understanding of the evolution of RNA editing in plant mitochondria.

## Methods

Forty-six taxa were included in this study, comprising the twelve families that form the core Alismatales. Seven taxa from Tofieldiaceae, Araceae, and Acorales are included in the outgroup (Tables S1 in Additional file 1). DNA extraction was performed using the plant DNA kit (Qiagen, Solvna, Sweden).

The data used in this study include five coding mitochondrial genes (*cob*, *atp*1, exon 2 of *nad*5, *mtt*2, and *ccm*B) comprising 4357 characters after alignment. Sequences of *cob *and *atp*1 were reused from Petersen et al. [28], the remaining were produced for this study. The PCR amplification primers used are shown in Table S5 in Additional file 1. Amplification reactions were performed in 50 μl total volume using about 50 ng of template DNA, 1 unit of Taq DNA polymerase (Ampliqon, Rødovre, Denmark), 40 pmol of each primer, 0.2 mM of each dNTP, 2.5 mM MgCl_2_, and 10× standard buffer provided by the company. The resulting PCR products were visualised on a 1% agarose gel and purified using QIAquick PCR Purification Kit (Qiagen). Purified PCR products were sequenced using ABI PRISM BigDye Terminator Cycle Sequencing v2.0 Ready Reaction Kit (AP Biosystems, Foster City, CA). Amplification primers were used as sequencing primers in *mtt*2 and *ccm*B, and additional internal primers were used to recover overlapping sequences for *nad*5, *cob *and *atp*1. Sequencing products were cleaned with Qiagen Dye-Ex Spin columns and analyzed on an ABI PRISM (PE Biosystems) automated DNA sequencer.

cDNA sequences were generated either from fresh plants or tissue stored in RNAlater (Qiagen). Total RNA was extracted using the Total RNA extraction kit (Ambion, Austin, TX) and DNAased with 1 μL of DNAase I (Promega, Madison, WI). The one step RT-PCR kit (Qiagen) was used to generate cDNAs and to amplify each region. Specific RNA primers were designed to anneal in the junction between the exon 1 and exon 2 of *nad*5 (Table S5 in Additional file 1). For the other genes in this study, the DNA primers were used. RT-PCR was performed in accordance to the protocol provided by the producer and using 50°C for 30 min. to generate the cDNA copies and 52°C as annealing temperature during the DNA amplification.

Edited sites were estimated using PREP-Mt [10]. This program works by contrasting the predicted amino acid sequence to the possible states of a codon with/without RNA editing against a homologous amino acid site of the coding gene from which RNA editing has been experimentally determined or from organisms with lack of editing capability. Results of PREP-MT were verified by comparison to the cDNA sequences that we generated (Table S2 in Additional file 1). For the purposes of this study, we considered a site as edited when at least one taxon showed an edited C at a particular position of the alignment, even though cDNA sequences were not obtained for all included taxa. All sites with experimental evidence of editing in some taxa but not edited in others were excluded from the analysis.

Phylogenetic reconstruction was carried out in PAUP* ver. 4.0b10 [43] using parsimony as the optimality criterion. The effect of edited sites in phylogenetic reconstruction is spurious, in particular if both homologous and processed paralogs are sampled. To minimize this potential source of error, we excluded the edited sites from the analyses. An initial heuristic search was carried out with 1000 replicates, holding a maximum of 10 trees per replicate. A random stepwise addition sequence was used to create the starting tree in each replicate. Branch swapping was performed using tree-bisection reconnection (TBR) with MulTrees in effect. A second search was performed by TBR-swapping the trees saved from the 1000 replicates in the first search. Bootstrap proportions were calculated based on 500 bootstrap replicates, using the same settings as for each replicate in the heuristic search, but with only 10 random stepwise addition sequences per replicate.

Maximum likelihood (ML) searches were performed using GARLI version 0.96b8 [44] with the general time-reversible model + invariant sites + gamma-distributed rate heterogeneity and the default settings for the search algorithm. Five initial runs of GARLI were carried out to ensure that the same topology was achieved.

In order to study the dynamics of loss and gain of edited sites during the evolution of the group, MacClade ver. 4.08 [45] was used to map edited sites. To do so, a matrix including only edited sites was constructed for each gene. Parsimony was used to reconstruct the states for each node of the phylogeny, and in the case of ambiguities in the mapping, they were resolved favoring parallel loss (DELTRAN) of edited sites over reversals (ACCTRAN). This decision is based on previous studies indicating that loses of edited sites are more probable that gains [19]. As a second approach, we predicted the amino acid sequence of the existing data and used PAML v. 4.2 [46] to reconstruct the ancestral amino acid sequence for each node in the phylogeny. This was done in codeml using the JTT amino acid substitution model [47]. Similarly, we reconstructed ancestral codon sequences. This was done assuming equal codon frequencies at equilibrium, equal amino acid distance, and treating *w *as a free parameter. We then compared the amino acid and codon sequence for each node, and tracked changes in edited sites on each branch. Both methods of character mapping gave similar results. The only difference was in the way a few characters are optimized at the base of the three. As these differences do not have a major impact on the character mapping, only the parsimony mapping will be considered in further analyses.

Synonymous branch lengths (d_S_) were estimated for each gene in HyPHy ver. 0.99 [48] using one parsimony tree and the MG94×HKY85_3×4 codon model with the local option in effect, allowing independent estimates for each branch of the tree. A pairwise relative rate test was used to estimate the probability of heterogeneity in the synonymous substitution rate between two taxa. This was also carried out in HyPhy using *Tofieldia *as outgroup.

In order to obtain the absolute synonymous substitution rate, a temporal scale is needed. To obtain relative ages of different clades, we use an age of 130 Ma to fix the age of Alismatales (*sensu *APGIII [29]), following previous monocot dating analyses based on *rbc*L [49]. Dating analyses were performed on the same topology as used to map edited sites. Because sequences of *ccm*B are missing for five taxa, this region was excluded from branch length calculations, together with the edited sites of the remaining regions. Estimates of branch lengths were obtained from the concatenated matrix by maximum likelihood optimization using the GTR+G+I model in PAUP*, with all parameters estimated from the dataset. Non-parametric rate smoothing as implemented in r8s [32] was used to obtain divergence times. Searches were restarted five times with a perturbation of the initial parameters to ensure that the solution reached a stable optimum.

The branch lengths were divided by duration time of to obtain an absolute value of synonymous substitutions per branch per gene (R_S_). However, as single gene d_S _values gave many branches with zero length, we used the distance to the most recent common ancestor (MRCA) of the core Alismatales divided by the age estimated for this node (79.1 Ma) to obtain a rough approximation of the absolute synonymous substitution rate for each taxon.

Changes in 3^rd ^codon positions were optimized on the tree using the same strategy as for optimization of edited sites. In this case, however, we did not decide *a priori *how to resolve ambiguities in optimization. C to T changes in 3^rd ^codon positions were counted in the same manner as changes in edited sites (e.g., by adding up the changes from all branches leading from the ancestor) in order to compare both estimates. Fisher exact test was used to assess whether the C-to-T changes were significant different in edited sites than in 3^rd ^codon positions.

Species are part of a hierarchical structured phylogeny, and cannot be considered as independent points in a regression analysis [35]. However, comparisons between sister clades allow us to test character correlations, because two sister branches are independent of each other after diverging. For each gene, except *atp*1, we calculated the d_S _distance from node-to-tip for each terminal branch in the tree. For branches deeper in the tree this was done by averaging the branch lengths from the daughter branches [33,34]. The same was done for the number of changes in edited sites for each branch. Independent contrasts were calculated as differences in branch lengths (d_S_) and number of changes in edited sites for sister clades descending from each node. As sister clades are of the same age, differences in their branch lengths directly reflect differences in their synonymous substitution rate, and the same applies to differences in rate of change in edited sites. We used a regression through the origin to explore the relationship [50] between d_S _and number of changes in edited sites. Additionally, we used Felsenstein's method of phylogenetically independent contrasts (PIC, [35]) as implemented in the PDAP package v. 1.14 [51] for Mesquite v. 2.72 [52]. In this analysis, contrasts were calculated for 1) the absolute synonymous substitution rate of each species (R_S_, shown in Table S4 in Additional file 1) and the number of changes in edited sites counted from the MRCA of the Alismatales to the tip of the tree, and 2) the R_S _and the number of edited sites present in each taxa. The PIC analyses were performed after standardizing for the age of the split among sister groups [53]. For each gene, the Pearson's correlation coefficient was calculated for each set of contrasts. In *nad*5 and *cob *many members of the Hydrocharitaceae have lost all their edited sites, and independent of the substitution rate, no more changes occur at these sites. In these cases, the PIC analyses were performed 1) including all taxa of Hydrocharitaceae, 2) removing the taxa of Hydrocharitaceae without edited sites but leaving the longest branch, and 3) removing the taxa of Hydrocharitaceae without edited sites but leaving the shortest branch.

## Authors' contributions

AC participated in the design of the study, carried out the laboratory work, performed the data analyses and wrote the paper. GP and OS obtained DNA samples, participated in the design of the study and in the manuscript writing. JID and DWS participated in the acquisition of DNA samples and in the manuscript writing. All authors have read and approved the final version of the manuscript.

## Supplementary Material

Additional file 1 **Supplementary tables**. Table S1 lists the taxon sampling, voucher and GenBank numbers for the DNA sequences used in this study. Table S2 lists the cDNA sequences used in study, voucher information and GenBank numbers. Table S3 indicates the number of edited sites per taxa (after the exclusion of sites where among taxa heterogeneity in editing status was found). Table S4 lists the absolute synonymous substitution rate (R_S_) in substitutions per billion year (79.11 MY), and Table S5 indicates the primers used in this study. Click here for file

Additional file 2 **Bootstrap tree from a combined analysis of five mitochondrial genes**. Click here for file
