# Supplementary material for: Are substitution rates and RNA editing correlated?
Source: BMC Evol Biol. 2010 Nov 11;10:349. doi: 10.1186/1471-2148-10-349 (PMC2989974; doi:10.1186/1471-2148-10-349)
Supplement: Additional file 1 — Supplementary tables. Table S1 lists the taxon sampling, voucher and GenBank numbers for the DNA sequences used in this study. Table S2 lists the cDNA sequences used in study, voucher information and GenBank numbers. Table S3 indicates the number of edited sites per taxa (after the exclusion of sites where among taxa heterogeneity in editing status was found). Table S4 lists the absolute synonymous substitution rate (RS) in substitutions per billion year (79.11 MY), and Table S5 indicates the primers used in this study. [file 1471-2148-10-349-S1.PDF]

Table S1 Taxon sampling and GenBank numbers for the DNA sequences used in this study

Family	Species	*cob*	*atp*1	*nad*5	*ccm*B	*mtt*2
Tofieldiaceae	*Pleea tenuifolia* Michx.	DQ916653	AY299827	DQ406995	HQ267387	HQ267427
	Tofieldia pusilla (Michx.) Pers.	DQ859122	HQ317981	HQ267471	HQ267386	HQ267426
Araceae	*Arisaema amurense* Maxim.	DQ859123	HQ317982	HQ267472	HQ267389	HQ267429
	Gymnostachys anceps R. Br.	DQ916647	AF039244	HQ267512	HQ267388	HQ267428
	Orontium aquaticum L.	DQ916648	AY299816	DQ406996	HQ267390	HQ267430
	Symplocarpus foetidus (L.) W. Barton	DQ978242	AF039245	HQ267473	HQ267391	HQ267431
Acoraceae	*Acorus calamus* L.	DQ859124	AF039256	HQ267474	-	HQ267432
Alismataceae	*Alisma plantago-aquatica* L.	DQ859125	AF197717	HQ267475	HQ267392	HQ267433
	Baldellia ranunculoides (L.) Parl.	DQ859127	DQ859092	HQ267478	HQ267395	HQ267436
	Caldesia oligococca (F. von Mueller) Buche	DQ859129	AY277800	HQ267480	HQ267396	HQ267439
	Echinodorus cordifolius (L.) Griseb.	DQ859132	DQ859096	HQ267482	HQ267398	HQ267441
	Echinodorus osiris Rataj	DQ859133	DQ859097	HQ267483	HQ267399	HQ267442
	Luronium natans (L.) Raf	HQ317976	HQ317983	HQ267492	HQ267407	HQ267450
	Ranalisma humile (Rich. ex Kunth) Hutch	HQ317977	HQ317984	HQ267502	HQ267416	HQ267460
	Sagittaria latifolia Willd.	Q978243	AY299832	HQ267513	HQ267418	HQ267462
Aponogetonaceae	*Aponogeton crispus* Thunb.	Q859126	Q859091	HQ267477	HQ267394	HQ267435
Butomaceae	*Butomus umbellatus* L.	DQ916649	AY299733	DQ406968	-	HQ267438
Cymodoceae	*Amphibolis griffithii* (J.M.Black) Hartog	HQ317978	HQ317985	HQ267476	HQ267393	HQ267434
	Cymodocea nodosa Asch.	DQ859130	DQ859094	HQ267481	HQ267397	HQ267440
	Syringodium isoetifolium(J.M.Black) Hartog	HQ317979	HQ317986	HQ267506	HQ267420	HQ267465
Hydrocharitaceae	*Blyxa aubertii* Rich.	DQ859128	DQ859093	HQ267479	-	HQ267437
	Egeria najas Planch.	DQ859134	DQ859098	HQ267484	HQ267400	HQ267443

	Elodea canadensis Michx.	DQ859135	DQ859099	HQ267485	HQ267401	-
	Halophila sp.	DQ859136	DQ859100	HQ267486	-	HQ267444
	Hydrilla verticillata (L. f.) Royle	DQ859137	DQ859101	HQ267488	HQ267403	HQ267446
	Najas guadalupensis (Sprengel) Magnus	DQ859143	DQ859107	HQ267493	HQ267408	HQ267451
	Najas sp.	DQ859144	DQ859108	HQ267494	HQ267409	HQ267452
	Nechamandra alternifolia (Roxb.) Thwaites	DQ859145	DQ859109	HQ267495	HQ267410	HQ267453
	Ottelia ovalifolia (R. Br.) Rich.	DQ859146	AY277802	HQ267496	-	HQ267454
	Stratiotes aloides L.	DQ859153	DQ859115	HQ267505	-	HQ267464
	Vallisneria sp.	DQ859158	DQ859119	HQ267509	HQ267423	HQ267468
Juncaginaceae	*Lilaea scilloides* (Poir.) Hauman	DQ859140	DQ859104	HQ267490	HQ267405	HQ267448
	Triglochin maritima L.	DQ859156	AY299852	HQ267507	HQ267421	HQ267466
	Triglochin palustris L.	DQ859157	DQ859118	HQ267508	HQ267422	HQ267467
Limnocharitaceae	*Hydrocleys nymphoides* (Willd.) Buchenau	DQ859139	DQ859103	HQ267489	HQ267404	HQ267447
	Limnocharis flava (L.) Buchenau	DQ859142	DQ859106	HQ267491	HQ267406	HQ267449
Posidoniaceae	*Posidonia australis* Hook. f.	DQ859148	DQ859111	HQ267498	HQ267412	HQ267456
	Posidonia oceanica (L.) Delile	DQ859149	DQ859112	HQ267499	HQ267413	HQ267457
Potamogetonaceae	*Potamogeton lucens* L.	DQ859150	DQ859113	HQ267500	HQ267414	HQ267458
	Potamogeton natans L.	DQ916651	AY299829	HQ267501	HQ267415	HQ267459
	Zannichellia palustris L.	DQ859159	DQ859120	HQ267510	HQ267424	HQ267469
Ruppiaceae	*Ruppia cirrhosa* (Petagna) Grande	DQ859151	DQ859114	HQ267503	HQ267417	HQ267461
Scheuchzeriaceae	*Scheuchzeria palustris* L.	DQ859152	AY277803	HQ267504	HQ267419	HQ267463
Zosteraceae	*Heterozostera tasmanica* (M.Martens ex Asch.) Hartog	HQ317980	HQ317987	HQ267487	HQ267402	HQ267445
	Phyllospadix scouleri Hook.	DQ859147	DQ859110	HQ267497	HQ267411	HQ267455
	Zostera marina L.	DQ859160	DQ859121	HQ267511	HQ267425	HQ267470

Table S2. cDNA sequences used in study: voucher information and GenBank numbers

Gene	Family	Taxa	Voucher	GenBank #
atp1	Butomaceae	*Butomus umbellatus*	C-2457 (C)	HQ317974
	Hydrocharitaceae	*Stratiotes aloides*	C-2459 (C)	HQ317973
ccmB	Hydrocharitaceae	*Elodea sp.*	C-2458 (C)	HQ317952
		Hydrilla verticillata (L. f.) Royle	C-2472 (C)	HQ317947
		Vallisneria sp.	C-2446 (C)	HQ317950
	Juncaginaceae	*Triglochin maritima*	C-2452 (C)	HQ317946
	Potomagetonaceae	*Potamogeton gramineus*	C-2461 (C)	HQ317948
		Zannichellia palustris	C-2454 (C)	HQ317951
	Ruppiaceae	*Ruppia cirrhosa* (Petagna) Grande	C-2455 (C)	HQ317953
	Scheuchzeriaceae	*Scheuchzeria palustris*	C-2566 (C)	HQ317949
	Zosteraceae	*Zostera marina*	C-2451 (C)	HQ317954
cob	Hydrocharitaceae	*Elodea nuttallii.*	C-2458 (C)	HQ317966
		Stratiotes aloides	C-2459 (C)	HQ317965
	Juncaginaceae	*Triglochin maritima*	C-2452 (C)	HQ317963
	Potomagetonaceae	*Potamogeton gramineus*	C-2461 (C)	HQ317964
nad5	Acoraceae	*Acorus gramineus* Sol.	C-2445 (C)	HQ317972
	Alismataceae	*Alisma plantago-aquatica*	C-2444 (C)	HQ317971
	Hydrocharitaceae	*Elodea nuttallii*	C-2458 (C)	HQ317969
		Vallisneria sp.	C-2446 (C)	HQ317967
	Potomagetonaceae	*Zannichellia palustris*	C-2454 (C)	HQ317968
	Ruppiaceae	*Ruppia cirrhosa* (Petagna) Grande	C-2455 (C)	HQ317975
	Zosteraceae	*Zostera marina*	C-2451 (C)	HQ317970
mtt2	Alismataceae	*Alisma plantago-aquatica*	C-2444 (C)	HQ317962
	Butomaceae	*Butomus umbellatus*	C-2457 (C)	HQ317959
	Hydrocharitaceae	*Stratiotes aloides*	C-2459 (C)	HQ317958
	Juncaginaceae	*Triglochin maritima*	C-2452 (C)	HQ317955
	Potomagetonaceae	*Potamogeton palustris*	C-2461 (C)	HQ317956
		Zannichellia palustris	C-2454 (C)	HQ317960
	Ruppiaceae	*Ruppia cirrhosa* (Petagna) Grande	C-2455 (C)	HQ317961
	Scheuchzeriaceae	*Scheuchzeria palustris*	C-2566 (C)	HQ317957

Table S3. Number of edited sites per taxa per gene

		*atp*1	*ccm*B	*cob*	*mtt*2	*nad*5
Alismataceae	*Alisma*	0	19	3	25	1
	Baldellia	0	19	3	25	1
	Caldesia	0	19	2	25	19
	Echinodorus	0	19	0	11	19
	Luronium	0	19	3	25	1
	Ranalisma	0	19	1	25	19
	Sagittaria	0	19	6	25	19
Limnocharitaceae	*Hydrocleys*	0	8	4	14	19
	Limnocharys	0	18	11	25	19
Cymodoceaceae	*Amphibolis*	3	40	12	26	14
	Cymodocea	3	41	12	26	14
	Syringodeum	3	41	12	26	14
Posidoniaceae	*Posidonia*	3	42	15	26	17
Ruppiaceae	*Ruppia*	2	35	11	24	10
Zosteraceae	*Heterozostera*	3	41	16	24	15
	Phyllospadix	3	41	16	24	15
	Zostera	3	40	16	24	15
Potamogetonaceae	*Potamogeton*	3	41	17	24	15
	Zanichellia	3	40	17	24	15
Juncaginaceae	*Lilaea*	3	39	18	26	16
	Triglochin	3	40	18	24	16
Scheuchzeriaceae	*Scheuchzeria*	3	43	17	25	19
Aponogetonaceae	*Aponogeton*	3	43	17	25	19
Butomaceae	*Butomus*	3	-	15	26	17
Hydrocharitaceae	*Blyxa*	0	-	0	25	17
	Egeria	0	36	0	21	2
	Elodea	0	37	0	-	2
	Halophila	0	-	0	1	0
	Hydrilla	1	38	0	13	0
	Najas	3	40	0	24	0
	Nechamandra	1	29	0	19	0
	Ottelia	2	-	0	25	16
	Stratiotes	2	-	15	27	16
	Vallisneria	1	29	0	19	0
Araceae	*Arisaema*	3	41	21	26	21
	Gymnostachys	3	41	22	28	22
	Orontium	3	42	20	28	22
	Symplocarpus	3	42	22	27	22
Tofieldiaceae	*Pleea*	2	42	20	27	22
	Tofieldia	2	40	20	27	22
Acoraceae	*Acorus*	0	-	2	10	15

Table S4. Absolute synonymous substitution rate (R_S) in substitutions per billion year for each gene using the age of the most common recent ancestor (79.11 MY)

		*atp*1	*ccm*B	*cob*	*mtt*2	*nad*5
Alismataceae	*Alisma*	4.39	0.83	1.27	1.05	0.26
	Baldellia	4.75	0.83	1.27	1.05	0.26
	Caldesia	4.77	1.25	1.22	1.14	0.17
	Echinodorus	5.58	1.67	1.10	1.75	0.25
	Luronium	4.60	0.83	1.22	1.05	0.30
	Ranalisma	4.34	1.10	1.16	1.05	0.13
	Sagittaria	5.75	0.83	1.45	1.05	0.23
Limnocharitaceae	*Hydrocleys*	3.42	2.27	1.33	1.69	0.17
	Limnocharys	4.22	1.66	1.50	1.05	0.21
Cymodoceaceae	*Amphibolis*	2.78	1.58	0.67	0.69	0.72
	Cymodocea	3.57	1.18	0.53	0.69	0.68
	Syringodeum	2.52	1.18	0.58	0.79	0.68
Posidoniaceae	*Posidonia*	2.43	0.38	0.41	0.79	0.60
Ruppiaceae	*Ruppia*	4.03	2.41	1.56	1.75	1.08
Zosteraceae	*Heterozostera*	3.60	2.39	0.58	1.19	0.52
	Phyllospadix	3.36	1.60	0.53	1.29	0.52
	Zostera	3.73	1.99	0.53	1.19	0.52
Potamogetonaceae	*Potamogeton*	4.94	0.78	0.64	1.00	0.64
	Zanichellia	4.19	1.18	0.87	1.40	0.80
Juncaginaceae	*Lilaea*	2.88	4.01	0.37	1.20	0.46
	Triglochin	2.78	0.78	0.35	1.20	0.55
Scheuchzeriaceae	*Scheuchzeria*	1.58	1.82	0.47	0.80	0.35
Aponogetonaceae	*Aponogeton*	1.30	1.60	0.47	0.61	0.46
Butomaceae	*Butomus*	1.51		0.52	1.53	0.24
Hydrocharitaceae	*Blyxa*	2.54		0.81	1.08	0.32
	Egeria	2.77	0.78	0.76	1.01	0.47
	Elodea	2.67	0.78	0.70		0.42
	Halophila	4.96		2.13	2.20	2.88
	Hydrilla	2.83	4.59	1.29	1.56	1.19
	Najas	3.20	2.40	1.50	1.76	1.26
	Nechamandra	2.40	2.40	0.87	1.19	1.22
	Ottelia	2.27		0.41	1.08	0.29
	Stratiotes	1.89		0.65	0.60	0.32
	Vallisneria	1.98	1.99	0.71	0.99	1.13

Table S5. Primers used to amplify *mtt*2, *ccm*B, *nad*5, and the cDNA copy of nad5

Primer name	Sequence 3' – 5'
*mtt*2	
*mtt*2_150F	TCT TGG TTT GAC ATG GTT TAC G
*mtt*2_115F	GTT CGA ATC CGT TYC GTT C
*mtt*2_760R	TCA CGA ACT TGT ACA TAC RAT GC
*mtt*2_787R	CAT TAY ACT CGT CCA GCC
*ccm*B	
*ccm*B_10F	GAA GTA AGG AAA TGA GAC GAC
*ccm*B_589R	CTA ATC GAG ACC RAA ATT GG
*nad*5 (DNA)	
*nad*5 F1	CCG TAG TNA TGT YAA TTG TGG
*nad*5 R1	TAT CCT ACA AAR AKA CTM CC
*nad*5 (cDNA)	
*nad*5_m1	TTG GGG CTT CTT TTT CGA TAG
