# Supplementary figures and images for: Are substitution rates and RNA editing correlated?
Source: BMC Evol Biol. 2010 Nov 11;10:349. doi: 10.1186/1471-2148-10-349 (PMC2989974; doi:10.1186/1471-2148-10-349)

Additional file 2 - Bootstrap tree from a combined analysis of five mitochondrial genes

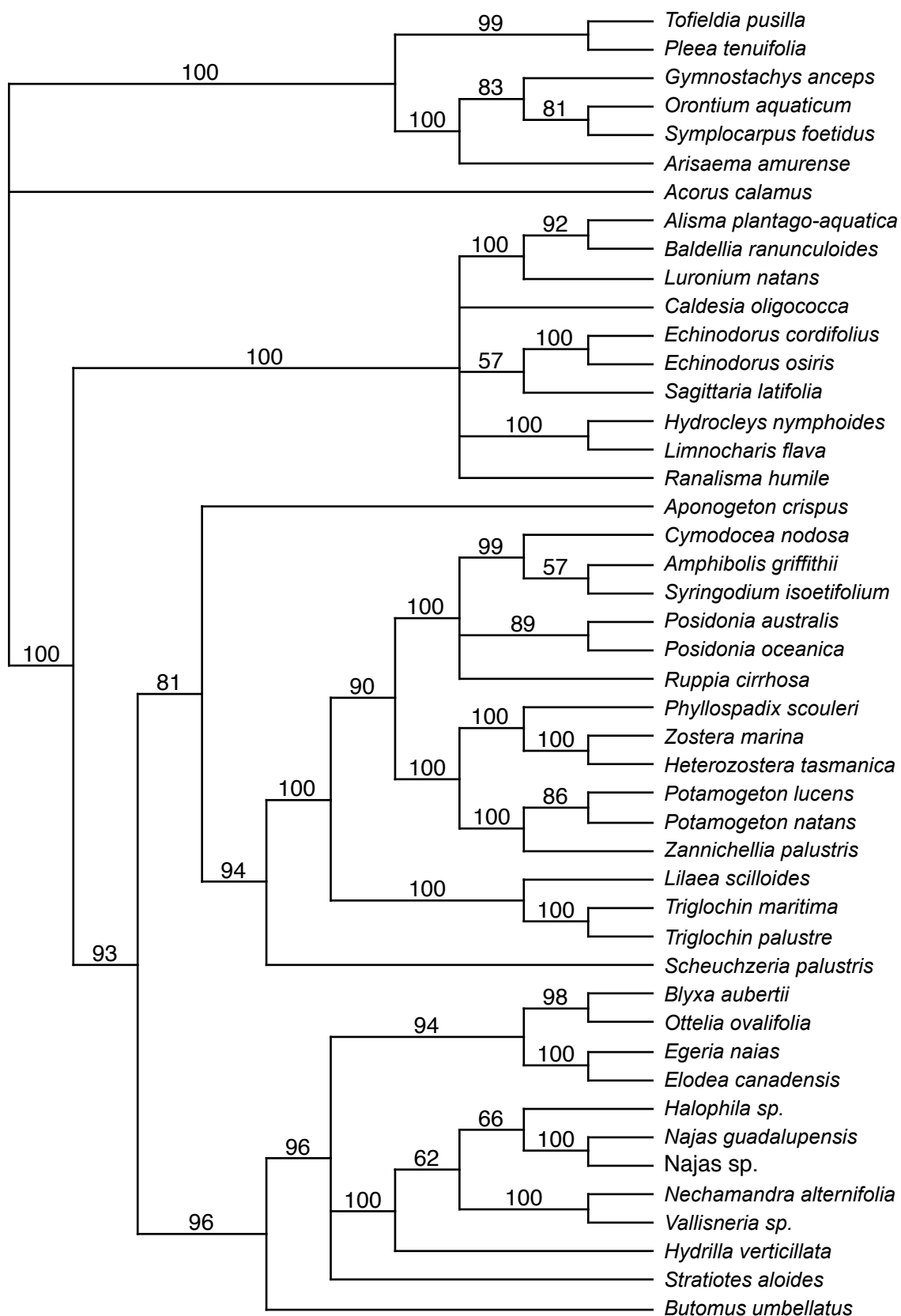

Supplement: Additional file 2 — Bootstrap tree from a combined analysis of five mitochondrial genes. [file 1471-2148-10-349-S2.PDF]
